# Pelvic Exenteration: An Ultimate Option in Advanced Gynecological Malignancies—A Single Center Experience

**DOI:** 10.3390/cancers17142327

**Published:** 2025-07-12

**Authors:** Helmut Plett, Jan Philipp Ramspott, Ibrahim Büdeyri, Andrea Miranda, Jalid Sehouli, Ahmad Sayasneh, Mustafa Zelal Muallem

**Affiliations:** 1 Department of Gynecology with Center for Oncological Surgery, Charité Medical University, 13353 Berlin, Germany; helmut.plett@charite.de (H.P.);; 2 Department of Obstetrics & Gynaecology, Caritas, Maria-Heimsuchung, Pankow, 13187 Berlin, Germany; 3 Department of General, Visceral and Transplant Surgery, University Hospital Muenster, 48149 Muenster, Germany; 4 Department of Gynecological Oncology, Surgical Oncology Directorate, Guy’s and StThomas’ NHS Foundation Trust, Faculty of Life Sciences and Medicine, School of Life Course Sciences, King’s College London, Westminster Bridge Road, London SE1 7EH, UK

**Keywords:** pelvic exenteration, Brunschwig’s operation, cervical cancer, vaginal cancer, uterine cancer, vulvar cancer, ovarian cancer, complex surgery, morbidity, mortality

## Abstract

**Background/Objectives**: Pelvic exenteration (PE) might be a curative option for patients with advanced pelvic malignancies. Due to its significant morbidity and mortality rates, PE necessitates meticulous patient selection, and a comprehensive understanding of disease spread. This study outlines the experience at a single tertiary referral center and investigates prognostic factors influencing survival post-PE, thereby guiding clinical decision-making processes. **Methods**: Patients undergoing PE for advanced pelvic gynecological malignancies between 01/2016 and 12/2023 were retrospectively analyzed using a prospectively managed database. Eligibility for PE was determined through individualized tumor board evaluations based on CT/MRI imaging, excluding patients with distant metastases. Baseline demographic and clinical characteristics, operative details, complication rates, and histopathological findings were assessed using univariate and multivariate regression analyses. Disease-free (DFS) and overall survival (OS) were assessed by Kaplan-Meier analysis. Poor outcome prognostic factors were identified, outlining an optimal candidate profile for PE. **Results**: A total of 70 patients were included. The median age was 54.5 years. Forty-three patients (61.4%) presented with recurrent disease and the majority were diagnosed with cervical cancer (n = 48, 68.6%). Total PE was performed in 40 patients (57.1%), with complete tumor resection achieved in 68.6% of patients (n = 48). Sixteen patients (22.8%) experienced grade IV/V complications. Median DFS and OS were 8.2 and 16.4 months, respectively. Multivariate analysis identified R1 resection status and para-aortic lymph node involvement as independent negative prognostic factors. **Conclusions**: PE is a viable option for selected patients with advanced primary and recurrent pelvic gynecological malignancies. When complete tumor resection is feasible, patients may derive benefit from PE, although the risk of severe perioperative complications must be carefully evaluated.

## Simple Summary

Pelvic exenteration is a complex surgical procedure primarily indicated for patients with advanced or recurrent gynecological malignancies, particularly recurrent cervical cancer. Achieving complete tumor resection necessitates a multi-visceral en-bloc resection of the affected pelvic gastrointestinal and genitourinary organs, followed by intricate reconstructive procedures. Despite its potential for long-term disease control, pelvic exenteration is associated with significant morbidity and mortality. Therefore, meticulous patient selection, a comprehensive understanding of the complex pelvic anatomy, and advanced surgical expertise are essential prerequisites. In this study, we aim to investigate prognostic factors associated with survival following pelvic exenteration. Identifying these predictors is crucial to optimizing patient selection, enhancing oncological efficacy, and minimizing perioperative complications.

## 1. Introduction

Pelvic exenteration (PE) is a viable therapeutic option for advanced gynecological malignancies, notably central pelvic cervical cancer recurrence without involvement of the pelvic sidewall, extrapelvic nodes, or peritoneal dissemination [1,2]. Cervical cancer ranks as the fourth most common cancer in women in terms of both incidence and mortality with an estimated 660,000 new cases and 350,000 deaths worldwide in 2022 [3]. In the early stages of cervical tumors, radical surgery, chemotherapy, and radiotherapy constitute the primary treatment modalities, and PE is not advised [1,2].

Initially characterized by Brunschwig in 1948 as a palliative intervention for recurrent cervical carcinoma, PE continues to be predominantly indicated for this purpose [4]. In addition to cervical cancer, PE may also be considered for locally advanced recurrences of endometrial [5,6] and vulvar cancer [7,8].

PE is a comprehensive multi-visceral surgical procedure aimed at achieving complete tumor resection by removing all affected pelvic organs. This procedure may involve the resection of gastrointestinal and genitourinary pelvic structures, including the distal sigmoid colon, rectum with anus, bladder with urethra, as well as the uterus, ovaries, and vagina. Although PE has traditionally been considered contraindicated in cases of lateral pelvic sidewall involvement, Höckel et al. introduced the concept of Laterally Extended Endopelvic Resection (LEER) to achieve complete resection in patients with lateral pelvic recurrence and locally advanced gynecological cancers [9,10]. In this approach, the pelvic resection plane is extended to encompass the medial portion of the acetabulum, obturator membrane, sacrospinous ligament, sacral plexus, and piriformis muscle, including the entire internal iliac vascular compartment [11,12].

PE may be significantly associated with postoperative morbidity following complex reconstructive procedures involving genitourinary, intestinal, and perineal structures [13,14]. Surgical complications in these cases may include anastomotic leaks, fistulas, and abscesses. Generally, complication rates following PE for all gynecological neoplasms range from 25% to 94%, and for cervical cancer from 25% to 83% [15,16,17,18,19,20,21,22,23,24,25,26].

The overall mortality rate for gynecological cancers is notably high at 25%, while the in-hospital mortality rate and 90-day mortality rate are reported as 2.65% and 2.74%, respectively [27].

The 5-year OS rate following PE ranges from 32% to 70% for all gynecological malignancies and 24% to 51% for cervical cancer [26].

PE, while potentially the optimal and occasionally the sole treatment intervention for advanced malignancies, is contentious because of the elevated morbidity rates and restricted OS. Moreover, the standardization of criteria and surgical methods for PE is highly challenging due to a diverse and limited patient group exhibiting various clinical presentations.

Further study is necessary to utilize survival markers and selection criteria for identifying appropriate candidates for PE, with the objectives of (1) reducing surgery-related problems and (2) enhancing cancer treatment outcomes.

Given the absence of prospective trials to date and the lack of evidence-based criteria for patient selection, our objective was to identify predictors that optimize patient selection, enhance oncological efficacy, and minimize perioperative complications.

## 2. Materials and Methods

### 2.1. Study Design and Ethical Approval

We performed a review of our prospectively managed database of patients who underwent PE between January 2016 and December 2023 at the Department of Gynecology, Charité Medical University, a tertiary European Society of Gynaecological Oncology (ESGO) referral center. This study was conducted in accordance with the Declaration of Helsinki, and the institutional review board of Charité Medical University approved this research (EA1/195/20). Prior to the collection of any clinical data, written informed consent was obtained from each patient.

### 2.2. Patient Selection

Medical records of all patients who underwent PE due to advanced ovarian, vulvar, vaginal, endometrial, or cervical cancer were reviewed. Eligibility for PE was determined through individualized tumor board evaluations based on CT/MRI imaging, and patients with distant metastases were excluded.

A total of 70 patients were identified, and all cases were included for the analysis of feasibility and morbidity. Fourteen patients were excluded from the survival analysis due to loss at follow-up and mortality resulting from complications within 90 days post-surgery, leading to a final cohort of 56 patients.

### 2.3. Data Collection

The parameters extracted and analyzed included age, prior therapies, date and type of surgery, type of reconstructive surgery, tumor entity, tumor stage, resection margins, lymphatic and vascular invasion, number and type of transfused blood products, duration of surgery, length of in-hospital stay, and, in cases of PE for relapsed disease, the time interval between the initial diagnosis and recurrence. Postoperative complications were classified according to the Clavien–Dindo classification [28].

### 2.4. Outcome Measures

The primary endpoints of this study were OS and DFS. OS was defined as the duration from the date of surgery to death from any cause. DFS was defined as the interval from surgery to the radiologically confirmed recurrence, as detected by either computed tomography (CT) or magnetic resonance imaging (MRI). Patients who did not experience these events were censored at the time of their last follow-up or at the conclusion of the observation period. In instances of loss at follow-up, the date of the last known and documented clinical status was utilized.

### 2.5. Statistical Analysis

Continuous variables are presented as the mean with the standard deviation (SD) if normally distributed or as the median with the interquartile range (25th–75th percentile) if not normally distributed or ordinal-scaled. Categorical variables are presented as absolute and relative frequencies (n, %). Time-to-event data were analyzed using the Kaplan–Meier method, and survival curves were compared using the log-rank test. The landmark method was applied to reduce immortal time bias. Univariate and multivariate analyses were performed using the Cox proportional hazards regression model. Based on multivariate results, two independent prognostic factors for OS were identified, allowing stratification into best- and worst-prognosis groups. All statistical tests were two-sided, and a *p*-value of ≤0.05 was considered statistically significant. Data analysis was conducted using R statistical software [29]. Some patients’ data from this present study have been partially included in the COREPEX study by Bizzarri et al. [30,31].

## 3. Results

### 3.1. Patient Cohort

Seventy consecutive patients who underwent PE between January 2016 and December 2023 met the study inclusion criteria. The median age was 54.5 years (range 32–84 years). The median body mass index was 23 kg/m^2^ (range 16–40 kg/m^2^). Most patients were classified as Eastern Cooperative Oncology Group (ECOG) 0/1 (n = 60, 85.7%), while ten patients (14.3%) were classified as ECOG 2. An equal proportion of patients in the study group were classified as American Society of Anesthesiologists (ASA) 2 and ASA 3, each of which comprises 45.7% of the cohort. Notably, five patients (7.1%) were diagnosed with a second malignancy.

The distribution of tumor entities was as follows: cervical (n = 48, 68.8%), endometrial (n = 8, 11.4%), ovarian (n = 6, 8.6%), vaginal (n = 5, 7.1%), and vulvar (n = 3, 4.3%).

The indication for exenteration was advanced primary disease in 27 patients (38.6%), and recurrent or persistent disease in 43 patients (61.4%). Ten patients presented after radical hysterectomy and colpectomy (14.3%), ten patients (14.3%) after radical hysterectomy and chemoradiation, six patients (8.6%) after radical hysterectomy and chemotherapy or radiation, and 22 (31.4%) patients after prior radiation or chemotherapy or chemoradiation. Almost one-third of patients (n = 22) had no prior therapy before PE (Table 1).

Six Patients (8.6%) suffered from recto- or vesicovaginal fistulas or both. Table 1 shows the patients’ and diseases’ characteristics.

### 3.2. Surgical Details

Total PE was performed in 40 patients (57.1%), anterior exenteration in 14 (20%), and posterior exenteration in 16 (22.9%). Additionally, a LEER procedure was required in 20 patients (28.6%) due to uni- or bilateral tumor extension. Similarly, due to tumor extension, 23 patients (32.9%) were operated infralevatorial and three patients (4.3%) translevatorial including resection of the piriformis muscle, os sacrum, and sacral plexus. A systematic pelvic lymphadenectomy was performed in 49 (70%) patients and a para-aortic lymphadenectomy in 42 (60%) patients. Three patients (4.3%) underwent unilateral ligation and resection of the external iliac vein, while two patients (2.9%) received unilateral ligation and resection of both the external iliac artery and vein. Additionally, six patients (8.6%) underwent bilateral ligation and resection of the internal iliac artery and vein, whereas five patients (7.1%) underwent the same procedure unilaterally.

The distribution of the reconstructive procedures was as follows: the most prevalent urinary diversion performed was a cutaneous ureterostomy, accounting for 31 patients (44.3%), followed by a urinary ileal conduit in 23 patients (32.9%). Two patients (2.9%) underwent vaginal reconstruction, including one case utilizing a uterus flap for the reconstruction of the posterior vaginal wall, and one omental J-flap [32,33]. Additionally, two patients (2.9%) underwent primary closure of the perineal wall using a rectus abdominis flap. An omental J-flap was employed in 11 (15.7%) patients to minimize surgical morbidity following a radical surgical procedure. The median duration of the surgical procedure was 307 min, with a range spanning from 157 to 652 min. Additionally, 44.3% (n = 31) of patients received intraoperative blood transfusions. Table 2 presents the surgical characteristics.

### 3.3. Perioperative Complications

Major complications (grade IIIa–IV) were identified in 29 (41.4%) cases. Four patients died due to major postoperative complications (Clavien–Dindo grade V). Two of these patients experienced fatal hemorrhagic events on postoperative days 8 and 9, respectively. The other two patients developed multi-organ failure subsequent to multiple revision procedures and passed away on postoperative days 29 and 33. Twenty-six patients underwent surgical revision laparotomy (37.1%). Eight patients (8.5%) experienced anastomotic insufficiency, eight patients (11.4%) had urinary leakage, ten patients (14.3%) presented with fascial dehiscence, eight patients (11.4%) developed intraabdominal abscesses, and nine patients (12.9%) suffered from postoperative hemorrhage. The 30-day mortality rate was 4.3%. The median length of hospital stay was 25 days (range 7–122 days). The complications are detailed in Table 3.

### 3.4. Histopathological Details

The primary histological types included squamous cell carcinoma (n = 48, 68.6%), adenocarcinoma (n = 15, 21.4%), and other types (n = 7, 10%). The median tumor diameter was 60 mm. Eleven patients (15.7%) presented with tumors exceeding 10 cm, while 13 patients (18.6%) had either undefined or unavailable tumor size data. Lymphatic invasion was observed in 35 patients (50%), and vascular invasion was identified in 15 patients (21.4%).

Complete tumor resection (R0) was accomplished in 48 patients, resulting in a complete resection rate of 68.6%. Positive lymphatic nodes were detected in 41 patients (58.8%), with 31 patients exhibiting positive pelvic nodes (44.3%) and 15 patients showing positive pelvic and para-aortic nodes (21.4%). Table 4 presents the histopathological details.

### 3.5. Survival and Risk Factors for Tumor Recurrence and Overall Survival

The median follow-up was conducted at 15 months. Thirty-seven patients (66.1%) experienced some kind of relapse and 29 patients (51.8%) died of the disease. The OS rate at three years was 34%, while the five-year OS rate was 20%. The survival outcomes of our cohort are detailed in Table 5. All risk factors considered for OS are presented in Table 6.

**Figure 1:**
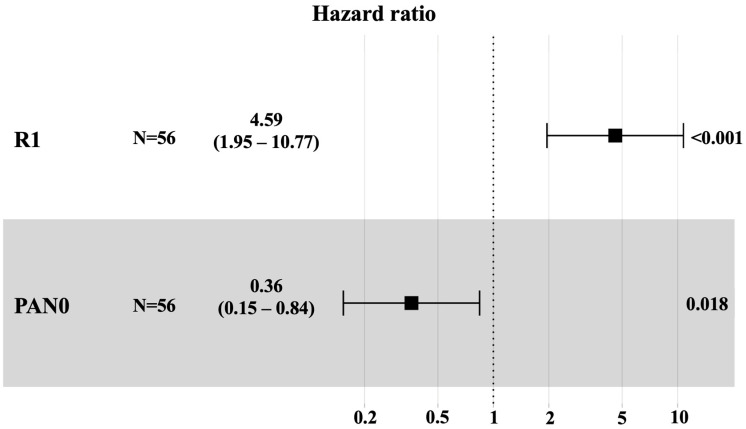
Multivariate Cox regression analysis for overall survival. Hazard ratios, associated confidence intervals, and *p*-values of positive resection margin (R1) and negative para-aortic lymph node (PAN) status are shown.

In univariate analysis, the most significant factors associated with worse OS were positive resection margins (R1) (HR 4.3; 95% CI 1.9–9.7; *p* < 0.001) and affected pelvic lymph nodes (HR = 2.3; 95% CI 1.1–5; *p* = 0.032). Non-affected para-aortic lymph nodes (HR = 0.46; 95% CI 0.22–0.99; *p* = 0.048) were associated with better OS. The multivariate Cox regression analysis revealed two independent prognostic factors that significantly impacted OS: positive resection margins (R1) were associated with worse OS, whereas negative para-aortic lymph node (PAN) status was associated with better OS (Figure 1).

**Figure 2:**
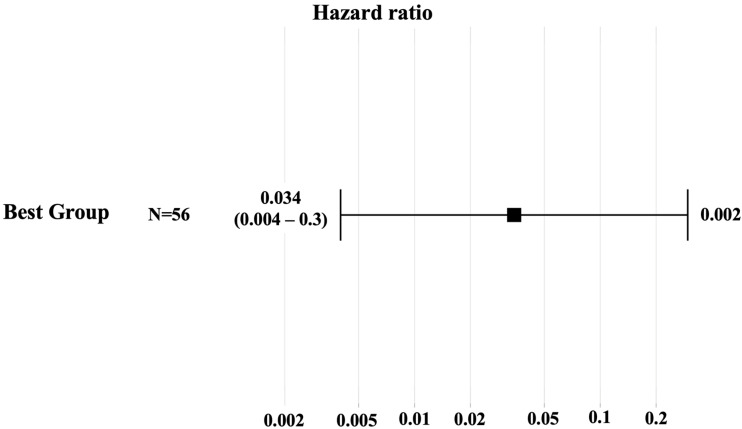
Forest plot of univariate Cox hazard analysis of the best group. Hazard ratio, associated confidence interval, and *p*-value are shown.

Patients exhibiting negative para-aortic lymph node status (PAN0) and clear resection margins (R0) demonstrated the most favorable outcome, characterized by a median OS of 52.5 months and a DFS of 9 months. This subgroup was thus deemed the most prognostically favorable cohort. Conversely, patients exhibiting both a positive resection margin (R1) and positive para-aortic lymph node status (PAN1) demonstrated the worst results, with a median OS of 14.9 months and a median DFS of 6.4 months. Consequently, this cohort was classified as the group with the most unfavorable outlook. The comparison of these two groups by Cox regression demonstrated a significantly diminished mortality risk in the best group (HR = 0.034; 95% CI: 0.004–0.3; *p* = 0.002), as illustrated in Figure 2.

**Figure 3:**
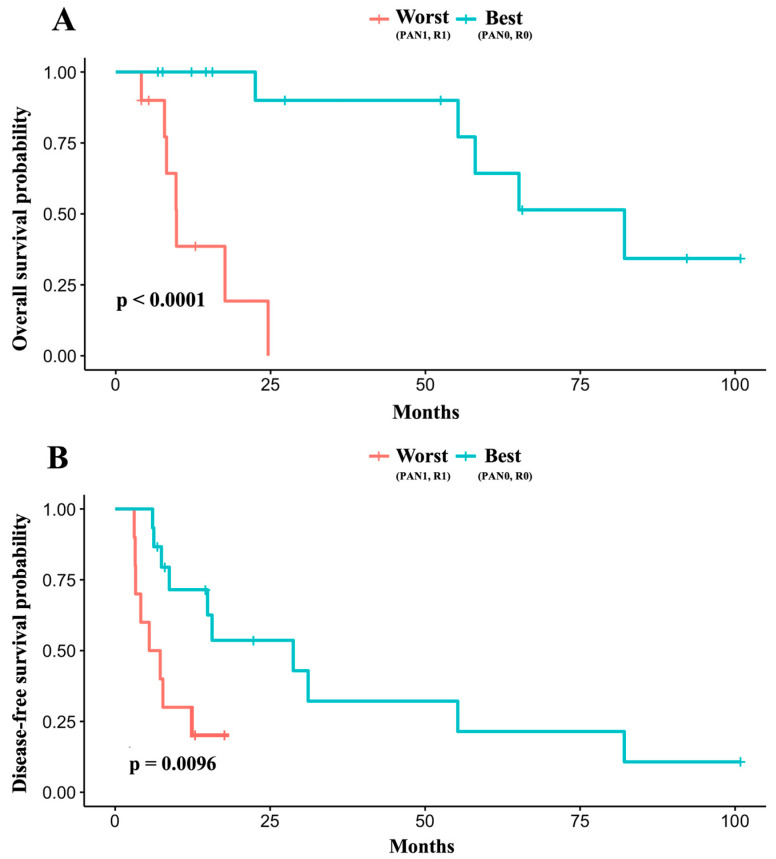
Comparison of (**A**) overall and (**B**) disease-free survival between the best (negative para-aortic lymph node status [PAN0] and clear resection margins [R0]) and the worst group (positive para-aortic lymph node status [PAN1] and positive resection margin [R1]). Survival curves were compared using the log-rank test. *p*-values are shown.

Kaplan–Meier analysis of these two groups further demonstrated significantly improved OS (*p* < 0.0001) and DFS (*p* = 0.0096) in the best group, as depicted in Figure 3.

## 4. Discussion

Since Brunschwig’s pioneering work in PE, in which he documented 24 cases of surgical intervention in patients with recurrent cervical cancer [4], PE continues to serve as a valuable therapeutic option for specific cases of gynecological cancer, particularly when all other treatment modalities have been exhausted. It is implemented globally in specialized cancer centers. However, this procedure requires a high level of surgical expertise and robust multidisciplinary collaboration. To date, there are no prospective trials available, and patient selection is not based on clear evidence. Therefore, it is crucial that these predictors optimizing patient selection, enhancing oncological efficacy, and minimizing perioperative complications be identified.

Recent studies have identified optimal resection margins (R0) as critical for improved survival outcomes [18,30,31,34], a finding we can corroborate with the institutional data in our study. Achieving an R0 resection has been consistently identified by most authors as a critical prognostic factor for enhancing overall survival. Consequently, increasing R0 resection rates should remain a primary objective for both oncologists and surgeons. In our cohort, we achieved an R0 resection rate of 68.6%, with an additional 7.1% classified as Rx. This aligns with the findings of Schmidt et al., who reported an R0 rate of 65% [18], and Egger et al., who reported a rate of 71.4% [34]. In our subgroup of R1 patients, we encountered four individuals with recurrent ovarian cancer (low-grade, high-grade, granulosa-cell) and one with sarcoma where complete resection was unattainable and tumor reduction was a secondary goal of palliative surgery, which may account for our “low rate” of R0 resection. With ongoing advancements in imaging quality and increasing surgical expertise, we anticipate that R0 resection rates could rise to 80% or even higher in the future.

Positive lymph node involvement is frequently identified as a risk factor for decreased OS [30,31]. They are not always apparent in preoperative MRI or CT scans, leading to the omission of systematic lymphadenectomy in certain situations. Our observations substantiate the concept of systematic lymph node excision in both the pelvic and para-aortic regions. Indeed, positive para-aortic nodes, in conjunction with clear margins, emerged as the second independent predictor for improved OS in the multivariable analysis. Consequently, we emphasize the significance of meticulous lymph node excision. We must not overlook para-aortic lymphatic illness, as previously noted by other authors in earlier investigations [30,31].

Although lymphatic invasion exhibited a tendency towards poorer outcomes, this finding did not reach statistical significance in our study (*p* = 0.069). In the COREPEX study, the authors identified the presence of lymphatic invasion as a significant factor independently associated with reduced OS [30]. Additionally, age was considered a substantial factor for improved survival [18,30,34]. Although we were unable to demonstrate its significance in our study (>65 years).

A total of 43 patients (61.4%) presented with recurrent disease and had previously undergone various treatments, including chemotherapy, radiotherapy, surgery, or a combination thereof.

Interestingly, despite concerns that previous exposure to cytotoxic or radiation-based therapies may compromise the subsequent treatment efficacy or overall OS, our data analysis revealed no statistically significant association between prior treatment and worse OS outcomes. This may suggest that prior therapeutic interventions may not reduce the potential for favorable survival. This emphasizes the need to consider an individualized treatment pathway within a broader clinical context rather than excluding patients based on historical therapy alone.

In our study, the independent parameters affecting OS are achieving a complete resection of the tumor, ensuring clear margins and the absence of malignancy in both pelvic and para-aortic lymph nodes.

Complication rates associated with this procedure are notably high due to increased intraoperative morbidity, complex reconstructive techniques, prolonged surgical duration, and significant blood loss, among other factors, to the point where the therapeutic potential of PE is outweighed by mortality and morbidity.

Morbidity is typically elevated in patients undergoing PE, with 30-day mortality rates reported at approximately 5.89%, as reported in a recent meta-analysis by Esmailzadeh et al. [27]. In our cohort of 70 patients, the 30-day mortality rate was 4.3%, which is slightly below the reported averages. Schmidt et al. reported a perioperative mortality rate of 5% in a cohort of 282 patients, although the specific causes of death were not disclosed [18]. Vigneswaran et al. documented a 12.8% return rate to the operating room due to complications and a 30-day mortality rate of 4% [35]. While large institutional experience might contribute to reduced morbidity and perioperative mortality, to our knowledge, no detailed data specifically addressing this relationship have been published to date. Such data would be valuable for future clinical guidance. Nonetheless, several studies have reported high morbidity and mortality rates during the early phases of implementing these procedures, both historically and in more recent times [4,36]. And even more critical factors such as tumor biology, surgical intent, the proportion of palliative exenterations, and the patient’s preoperative morbidity must also be taken into account when interpreting morbidity and mortality outcomes in this context. Mortality increased to 17.1% at 90 days in our study, underscoring the considerable cumulative risk during the postoperative period. In this context, nearly half of the patients undergoing this radical procedure required at least one secondary surgical intervention. Therefore, the management of this type of surgery is not solely defined by the primary intervention but also significantly relies on effective complication management. Reducing complication rates remains a primary objective in PE and involves identifying vulnerable patients for whom extensive surgery may ultimately be detrimental. Macciò et al. investigated the quality of life following pelvic evisceration, utilizing the European Organization for Research and Treatment of Cancer, Quality of Life (EORTC-QoL) questionnaire with close postoperative follow-up. Their results indicated an enhancement in spiritual well-being, particularly at one and three months postoperatively, underscoring the significance of offering psychological and spiritual support to patients and their families after PE [37].

The primary limitation of this study is its retrospective design, which may increase the likelihood of selection bias and reduce the ability to verify causality. Second, missing data and follow-up entries may have an impact on the strength of the statistical analysis. Third, the study was conducted at a single tertiary facility, which may limit the findings’ application to other institutions with varying levels of surgical skills and resources. Lastly, the heterogeneity of tumor kinds, stages, prior treatments, and adjuvant treatments in the data may have resulted in survival bias, making it difficult to draw clear conclusions about the oncological impact of surgical exenteration in enhancing survival.

However, the main strength of this study is its extensive institutional review conducted over a relatively brief inclusion period of nine years, which renders this cohort distinctive and facilitates the derivation of robust conclusions. Utilizing our centralized database, the study systematically identified detailed surgical events.

In the context of personalized medicine, where an increasing array of treatment options is available, particularly in the era of immunotherapy and antibody-drug conjugates, the availability of more options is undoubtedly beneficial for patients. The identification of relevant mutations through genome sequencing will undoubtedly lead to novel personalized treatment modalities. Nevertheless, surgery continues to play a crucial role and, in many instances, remains the sole viable option. This study aims to elucidate the complex pathway of PE management, from decision-making to the handling of complications, and may be regarded as an insightful examination of this experience, offering valuable lessons from a tertiary referral cancer center.

## 5. Conclusions

PE is a viable and potentially curative surgical option for meticulously selected patients with advanced or recurrent gynecologic cancers, contingent upon its execution in specialized high-volume centers with multidisciplinary expertise, which is also crucial for managing complications.

Notwithstanding the substantial morbidity, our findings underscore the prognostic importance of attaining complete (R0) resection and accentuate the necessity of thorough lymphadenectomy, especially in the para-aortic region, to prevent understaging and enhance survival outcomes. These criteria must be meticulously evaluated during preoperative planning to inform patient selection and mitigate unnecessary risk. Considering the significant incidence of postoperative problems and reinterventions, future initiatives should concentrate on enhancing perioperative treatment and improving eligibility criteria to more accurately identify patients who are most likely to benefit from this extreme operation. Additionally, the role of psychological and social support in enhancing the quality-of-life post-surgery should be emphasized. Future investigations are essential to provide effective selection algorithms that incorporate tumor biology, anatomical extent, and individual patient risk profiles, particularly considering the advancing significance of immunotherapy and targeted systemic therapies.

Future prospective multicenter trials are essential to refine patient selection criteria based on tumor biology, disease history, and anatomical extent, ensuring a more evidence-based and individualized treatment strategy.

## Figures and Tables

**Table 1:** Patient and disease characteristics. ASA: American Society of Anesthesiologists, ECOG: Eastern Cooperative Oncology Group, BMI: Body Mass Index.

Variable	Total n (%)
n (%)	70 (100%)
Age, median (range), years	54.5 (32–84)
ASA-Score	
ASA 1	4 (5.7%)
ASA 2	32 (45.7%)
ASA 3	32 (45.7%)
ASA 4	2 (2.9%)
ECOG	
0/1	60 (85.7%)
2	10 (14.3%)
2nd malignancy	5 (7.1%)
BMI, median (range), kg/m^2^	23 (16–40)
Tumor entity	
Cervical cancer	48 (68.6%)
Endometrial cancer	8 (11.4%)
Ovarian cancer	6 (8.6%)
Vaginal cancer	5 (7.1%)
Vulvar cancer	3 (4.3%)
Primary diagnosis	27 (38.6%)
Recurrent/persistent disease	43 (61.4%)
Surgery/other therapy prior to pelvic exenteration	
Radical hysterectomy/colpectomy	10 (14.3%)
Radical hysterectomy and chemoradiation/chemotherapy/radiation	16 (22.9%)
Radiation/chemotherapy/chemoradiation	22 (31.4%)
No therapy before PE	22 (31.4%)

**Table 2:** Surgical characteristics. LEER: Laterally Extended Endopelvic Resection.

Variable	Total, n (%)
n (%)	70 (100%)
Duration of surgery, median (range), min.	307 (157–652)
Type of pelvic exenteration	
Anterior	14 (20%)
Posterior	16 (22.9%)
Complete	40 (57.1%)
LEER procedure (total)	20 (28.6%)
Anterior, lateral extended	4 (5.7%)
Posterior, lateral extended	4 (5.7%)
Complete, lateral extended	12 (17.1%)
Level of pelvic exenteration	
Supralevatorial	44 (62.9%)
Infralevatorial	23 (32.9%)
Translevatorial	3 (4.3%)
Resection of anus	11 (15.7%)
Resection of vulva (radically/partially)	13 (18.6%)
Type of reconstruction	
Ileal conduit	23 (32.9%)
Ureterocutaneostomy	31 (44.3%)
Colostomy	43 (61.4%)
Jejuno-/ileostomy	2 (2.9%)
Vaginal reconstruction	2 (2.9%)
Use of flap	
Rectus abdominis flap	2 (2.9%)
Uterus flap for vaginal reconstruction	1 (1.4%)
Omental J-flap	11 (15.7%)
Additional surgical steps	
Ligation/resection of V./A. iliaca externa	5 (7.1%)
Ligation/resection of V./A. iliaca interna	11 (15.7%)
Neural resection	1 (1.4%)
Nerve-sparing procedure	14 (20.0%)
Patients receiving intraoperative blood products	31 (44.3%)
Number of red cell units transfused, median (range)	2 (1–27)
Number of fresh-frozen plasmas transfused, median (range)	3 (1–49)

**Table 3:** Peri- and postoperative complications.

Variable	Total, n (%)
n (%)	70 (100%)
Length of hospital stay, median (range), days	25 (7–122)
Postoperative complications	
Postoperative hemorrhage	9 (12.9%)
Surgical revision	26 (37.1%)
Postoperative infection	34 (48.6%)
Intraabdominal abscess (revision surgery)	8 (11.4%)
Wound dehiscence	16 (22.9%)
Fascial dehiscence	10 (14.3%)
Anastomotic insufficiency	6 (8.5%)
Urinary leakage	8 (11.4%)
Neural impeachment	1 (1.4%)
Multiorgan failure	4 (5.7%)
Renal failure	9 (12.9%)
Lower limb ischemia	2 (2.9%)
Clavien–Dindo classification	
I	23 (32.9%)
II	18 (25.7%)
IIIa	3 (4.3%)
IIIb	10 (14.3%)
IVa	7 (10%)
IVb	5 (7.1%)
V	4 (5.7%)

**Table 4:** Histopathological details.

Variable	Total, n (%)
n (%)	70 (100%)
Type of histology	
Squamous cell carcinoma	48 (68.6%)
Adenocarcinoma	15 (21.4%)
Other	7 (10%)
Tumor size, median (range), mm	60 (1–170)
Tumor infiltration	
Bladder	39 (55.7%)
Rectum	34 (48.6%)
Bladder and rectum	21 (30%)
Lymphatic invasion	
L0	24 (34.3%)
L1	35 (50%)
Lx ^1^	11 (15.7%)
Vascular invasion	
V0	44 (62.9%)
V1	15 (21.4%)
Vx ^1^	11 (15.7%)
Nodal Status	
N0	23 (32.9%)
N1	41 (58.8%)
Nx	6 (8.6%)
R-Status	
R0	48 (68.6%)
R1	17 (24.3%)
Rx	5 (7.1%)
Grading	
G2	19 (27.1%)
G3	39 (55.7%)
Gx ^1^	12 (17.1%)

^1^ Unavailable data due to tumor necrosis or condition after radio- or chemotherapy.

**Table 5:** Adjuvant therapy and survival data. OS: Overall Survival, DFS: Disease-Free Survival.

Variable	Total, n (%)
n (%)	56 (80%)
Therapy after pelvic exenteration	
Wait and see	34 (60.7%)
Chemotherapy	19 (33.9%)
Radiation	3 (5.4%)
Chemoradiation	0 (0%)
Follow up, median (range), months	15 (4–100)
Relapse of disease	37 (66.1%)
Dead of disease	29 (51.8%)
30-d mortality	4.3%
Median estimated DFS (months)	8.2
Median estimated OS (months)	16.4
Three-year OS	34%
Five-year OS	20%

**Table 6:** Univariate analysis of risk factors for overall survival. Bold *p*-values are statistically significant. ASA: American Society of Anesthesiologists.

Variable	N (%)	HR (95% CI)	*p*-Value
Total	56 (80%)		
Age			0.08
<65	47	1	
≥65	9	2.8 (0.74–7.8)	
ASA status			0.808
1/2	31	1	
3/4	25	1.1 (0.64–1.8)	
Diagnosis			
Cervical cancer	36	0.92 (0.38–2.2)	0.858
Vulvar cancer	3	0.5 (0.08–3.8)	0.334
Endometrial cancer	7	0.64 (0.19–2.1)	0.468
Ovarian cancer	6	1.6 (0.36–7.3)	0.526
Vaginal cancer	4	4.4 (0.97–20)	0.055
Presentation at diagnosis			0.084
Primary diagnosis	21	1	
Recurrent disease	35	0.46 (0.19–1.1)	
Therapy prior PE			
Chemotherapy	26	1.1 (0.49–2.3)	0.882
Radiotherapy	21	0.58 (0.27–1.2)	0.158
Chemoradiation	15	0.76 (0.36–1.6)	0.472
Surgical characteristics			
Duration of surgery, min.			0.46
<300	29		
≥300	27	1 (0.99–1)	
Type of Pelvic exenteration			
Anterior	11	1.5 (0.61–3.8)	0.366
Posterior	13	1.3 (0.54–3.4)	0.519
Complete	32	1.7 (0.8–3.7)	0.161
Clavien–Dindo classification			0.398
<IIIa	36	1	
≥IIIa	20	1.4 (0.64–3)	
Ileal conduit			0.267
No	38	1	
Yes	18	1.5 (0.72–3.2)	
Type of histology			
Squamous cell	37	1.5 (0.6–3.7)	0.388
Adenocarcinoma	12	0.48 (0.14–1.6)	0.237
Other	7	1.2 (0.35–4.1)	0.779
Lymphatic invasion			0.069
L0	18	1	
L1	28	2.7 (0.92–8.1)	
Nodal status			
N0	26	0.5 (0.23–1.1)	0.075
N1	28	1.8 (0.83–3.7)	0.144
Nx	2	8.2 (0.95–71)	0.055
Para-aortic lymph node status			
N0	22	0.46 (0.22–0.99)	**0.048**
N1	11	1.1 (0.44–2.7)	0.862
Nx	23	2.3 (1.1–4.9)	0.034
Pelvic lymph node status			
N0	26	0.71 (0.34–1.5)	0.362
N1	25	2.3 (1.1–5)	**0.032**
Nx	5	0.35 (0.082–1.5)	0.165
R-status			**<0.001**
R0	38	1	
R1	14	4.3 (1.9–9.7)	
Systematic pelvic lymphadenectomy			0.762
No	19	1	
Yes	37	1.1 (0.53–2.4)	
Pelvic lymphadenectomy (systematic/bulky/sentinel)			0.165
No	5	1	
Yes	51	2.8 (0.65–12)	
Systematic para-aortic lymphadenectomy			0.278
No	29	1	
Yes	27	0.67 (0.32–1.4)	
Para-aortic lymphadenectomy (systematic/sampling)			**0.034**
No	23	1	
Yes	33	0.44 (0.2–0.94)	
Adjuvant therapy			
Wait and see	34	0.71 (0.34–1.5)	0.368
Chemotherapy/radiation	22	1.4 (0.67–2.9)	0.368

## Data Availability

Data supporting results are housed in an in-hospital database. Regulatory issues do not allow the provision of a link to analyzed data sets.

## Abbreviations

The following abbreviations are used in this manuscript:

PE	Pelvic Exenteration
LEER	Laterally Extended Endopelvic Resection
ESGO	European Society of Gynaecological Oncology
OS	Overall Survival
DFS	Disease-Free Survival
CT	Computed Tomography
MRI	Magnetic Resonance Imaging
SD	Standard Deviation
CI	Confidence Interval
PAN	Para-aortic Lymph Node
EORTC-QoL	European Organization for Research and Treatment of Cancer, Quality of Life
